# Are social inequalities in acute myeloid leukemia survival explained by differences in treatment utilization? Results from a French longitudinal observational study among older patients

**DOI:** 10.1186/s12885-019-6093-3

**Published:** 2019-09-05

**Authors:** Eloïse Berger, Cyrille Delpierre, Fabien Despas, Sarah Bertoli, Emilie Bérard, Oriane Bombarde, Pierre Bories, Audrey Sarry, Guy Laurent, Christian Récher, Sébastien Lamy

**Affiliations:** 1 LEASP, UMR 1027, Equipe labellisée Ligue Nationale Contre le Cancer, Faculté de médecine de Purpan, Inserm-Université Toulouse III Paul Sabatier, 37 allées Jules Guesde, 31000 Toulouse, France; 2 Service de Pharmacologie Clinique, CHU de Toulouse, Toulouse, France; 3 Service d’hématologie, Institut Universitaire du Cancer de Toulouse - Oncopôle, CHU de Toulouse, Toulouse, France; 4 Service d’Epidemiologie, CHU de Toulouse, Toulouse, France; 5 Réseau régional de cancérologie Onco-Occitanie, Institut Universitaire du Cancer de Toulouse Oncopôle, Toulouse, France; 6 Centre de Recherche en Cancérologie de Toulouse UMR 1037 Inserm / ERL5294 CNRS, University of Toulouse 3 Paul Sabatier, Toulouse, France

**Keywords:** Acute myeloid leukemia, Observational study, French European deprivation index, Cancer management and survival, Elderly patients

## Abstract

**Background:**

Evidences support social inequalities in cancer survival. Studies on hematological malignancies, and more specifically Acute Myeloid Leukemia (AML), are sparser. Our study assessed: 1/ the influence of patients’ socioeconomic position on survival, 2/ the role of treatment in this relationship, and 3/ the influence of patients’ socioeconomic position on treatment utilization.

**Methods:**

This prospective multicenter study includes all patients aged 60 and older, newly diagnosed with AML, excluding promyelocytic subtypes, between 1st January 2009 to 31st December 2014 in the South-West of France. Data came from medical files. Patients’ socioeconomic position was measured by an ecological deprivation index, the European Deprivation Index. We studied first, patients’ socioeconomic position influence on overall survival (*n* = 592), second, on the use of intensive chemotherapy (*n* = 592), and third, on the use of low intensive treatment versus best supportive care among patients judged unfit for intensive chemotherapy (*n* = 405).

**Results:**

We found an influence of patients’ socioeconomic position on survival (highest versus lowest position HR_Q5_: 1.39 [1.05;1.87] that was downsized to become no more significant after adjustment for AML ontogeny (HR_Q5_: 1.31[0.97;1.76] and cytogenetic prognosis HR_Q5_: 1.30[0.97;1.75]). The treatment was strongly associated with survival. A lower proportion of intensive chemotherapy was observed among patients with lowest socioeconomic position (OR_Q5_: 0.41[0.19;0.90]) which did not persist after adjustment for AML ontogeny (OR_Q5_: 0.59[0.25;1.40]). No such influence of patients’ socioeconomic position was found on the treatment allocation among patients judged unfit for intensive chemotherapy.

**Conclusions:**

Finally, these results suggest an indirect influence of patients’ socioeconomic position on survival through AML initial presentation.

**Electronic supplementary material:**

The online version of this article (10.1186/s12885-019-6093-3) contains supplementary material, which is available to authorized users.

## Background

Many studies support that social inequalities may exist at all steps of cancer care pathway, from the early stages of cancer development to survival [1–5]. Patients’ socioeconomic position (SEP)-related differences in stage at diagnosis and access to treatment have been pointed out as the most important explanatory factors of social inequalities in mortality. However, results may vary depending on the healthcare system specificity as, for instance, people in a public tax-supported healthcare setting would be less exposed to financial barrier to care than in private funding healthcare settings [1, 6]. In most cases, studies concerned solid tumors and very few papers have focused on hematological malignancies More specifically, studies dealing with the influence of SEP on acute myeloid leukemia (AML) care and outcome are sparser. In the USA, i.e. in a health system mainly based on private funding, ethnicity, insurance status, educational level, and income were found to affect overall survival [7–10], at least partially through SEP-related inequality in treatment utilization, mainly access to intensive therapy and hematopoietic stem cell transplantation [7, 8, 10–13]. In Scandinavia, where healthcare services are mainly public or tax-supported, studies supported an association between overall survival and SEP measured by occupational class [14], and education level [15] although this relationship was not observed systematically. Regarding SEP-related differences in treatment utilization, results differed from those observed in private funding healthcare setting [15], with a lower use of intensive therapy in the lower educational level group but only among older AML patients. This indicates that, in addition to the healthcare system, the influence of patients’ SEP on AML treatment and outcome may involve different mechanisms depending on patients’ age. Incidence of AML increases sharply with age and standard care regimens for older AML patients are based primarily on three perspectives: (1) intensive chemotherapy, which are toxic but curative; (2) hypomethylating agents as semi-palliative but active approach and (3) best supportive care. To our knowledge, study assessing the influence of SEP on treatment utilization, especially among older patients, only focused on the use of intensive therapy. In response to this, the present study aims at studying: 1/ the influence of patients’ SEP on survival, 2/ the role of treatment in this relationship, and 3/ the influence of patients’ SEP on treatment utilization using a prospective AML database from the multicentric oncology network Onco-Occitanie in the Southwest of France. Here, SEP-related differences in the choice of treatment are assumed to be a potential explanatory mechanism of SEP-related differences in survival.

## Methods

### Study design

The IUCT-O AML study is a prospective longitudinal study including all patients treated for an AML in the Midi-Pyrénées region in South-West of France (about 2.8 million of inhabitants) [16]. Patients diagnosed with AML are referred by personal physicians, primary care centers or directly, in the Leukaemia unit of the Toulouse University Hospital. Data are centralized at the University Hospital and recorded each week according to guidelines from the oncology healthcare network of the Midi Pyrenees region (ONCOMIP) [17]. The IUCT-O AML database is registered at the Commission Nationale de l’Informatique et des Libertés (CNIL) under N°1,778,920. We included all patients aged 60 and older, newly diagnosed with an AML, excluding M3- subtypes, diagnosed between 1st January 2009 to 31st December 2014.

### Data collection

Clinical data were collected from patients’ medical files and certified by the Data Management Committee of the anonymized AML database of Toulouse University Hospital. Patients yielded written inform consent allowing the collection of personal clinical and biological data in an anonymized database. In accordance to the declaration of Helsinki, the study was reviewed and approved by the research ethics committee at Toulouse University Hospital. Regarding patients’ outcome, we considered the time between diagnosis and death from all cause. Patients’ were followed up to May 2017. The maximum length of follow-up was 6 years and 8 months and half of the sample was followed at least 4 months. Treatment were categorized as intensive chemotherapy (IC), low intensity therapy (LIT) and best supportive care (BSC). LIT and BSC were considered as non-intensive therapy. Intensive chemotherapy regimen as well as treatment with hypomethylating agents has been described elsewhere [16, 18]. Due to the lack of individual SEP measures in medical record, we used an ecological-level measure of SEP to approach the patients’ individual situation from the geographical coordinates of their addresses at the time of diagnosis. The French version of the European Deprivation Index (EDI) was developed to assess social deprivation [19], built from the Townsend’s definition of deprivation as “a state of observable and demonstrable disadvantage relative to the local community or the wider society to which an individual, family or group belongs” [20]. For each address, we identify the geographical area of about 2000 inhabitants (IRIS) for which EDI was available. We consider the national quintile of EDI: living in the fifth quintile meant to live in an area belonging to the 20% most deprived areas in France. In addition, patients’ characteristics included age, sex, comorbidity assessed by the Charlson cormorbidity index (cci = 0, 1, or ≥ 2) [21] and performance status. The disease characteristics included white blood cell count (sample distribution tercile), AML ontogeny, i.e. de novo vs. secondary AML (including post-myelodysplastic syndrome AML, post-chronic myelomonocytic AML, post-myeloproliferative disorder AML and therapy-related AML), and cytogenetic prognosis was defined according to the refined British MRC classification [22].

### Statistical analysis

We use a theory-driven approach to study whether patients’ SEP affect survival directly or through potential intermediate factors. In response to our two first objectives (step 1 analysis), we tested the influence of patients’ SEP on overall survival (objective 1) and the effect of the adjustment for treatment on the SEP – survival relationship (objective 2). We used Cox models with time-varying component for survival analyses to correct for non-proportional hazards. Then, we focused on the influence of patients’ SEP on the treatment received. As previously suggested by Bories et al., we assumed induction treatment choice to be a 2-steps process. First, the patients’ fitness for IC is assessed (step 2 analysis). Then, among those judged unfit for IC, the fitness for LIT is assessed (step 3 analysis). Accordingly, we built a two-step analysis testing for SEP-related differences in 1/ receiving IC or not among all patients, and 2/ receiving LIT or BSC among patients judged unfit for IC. We built generalized linear models estimating the probability of receiving 1/ IC (versus LIT or BSC), and 2/ LIT (versus BSC) as a function of EDI quintile (ref: the less deprived quintile (quintile 1)). Covariates were entered in models, first alternatively, and then simultaneously to assess potential intermediate variables in the pathway linking patients’ SEP to survival and treatment. All models were systematically adjusted for age, sex, and comorbidity. Potential confounders were identified from bivariate analyses as being associated with the outcomes, i.e. the death from all cause or the selected treatment. We fixed type I errors threshold to 0.2 and 0.05 for respectively bivariate and multivariable analyses. In sensitivity analysis we used multiple imputation methods for dealing missing data on both patients’ SEP and confounders [23, 24]. Imputation models were based on the available information regarding patients’ age, sex, performance status, AML ontogeny, level of white blood cells, and also the treatment received [25]. All analyses were done by using STATA release 14 (StataCorp LP, College Station, TX, USA).

## Results

### Selection of the study population

**Fig. 1:**
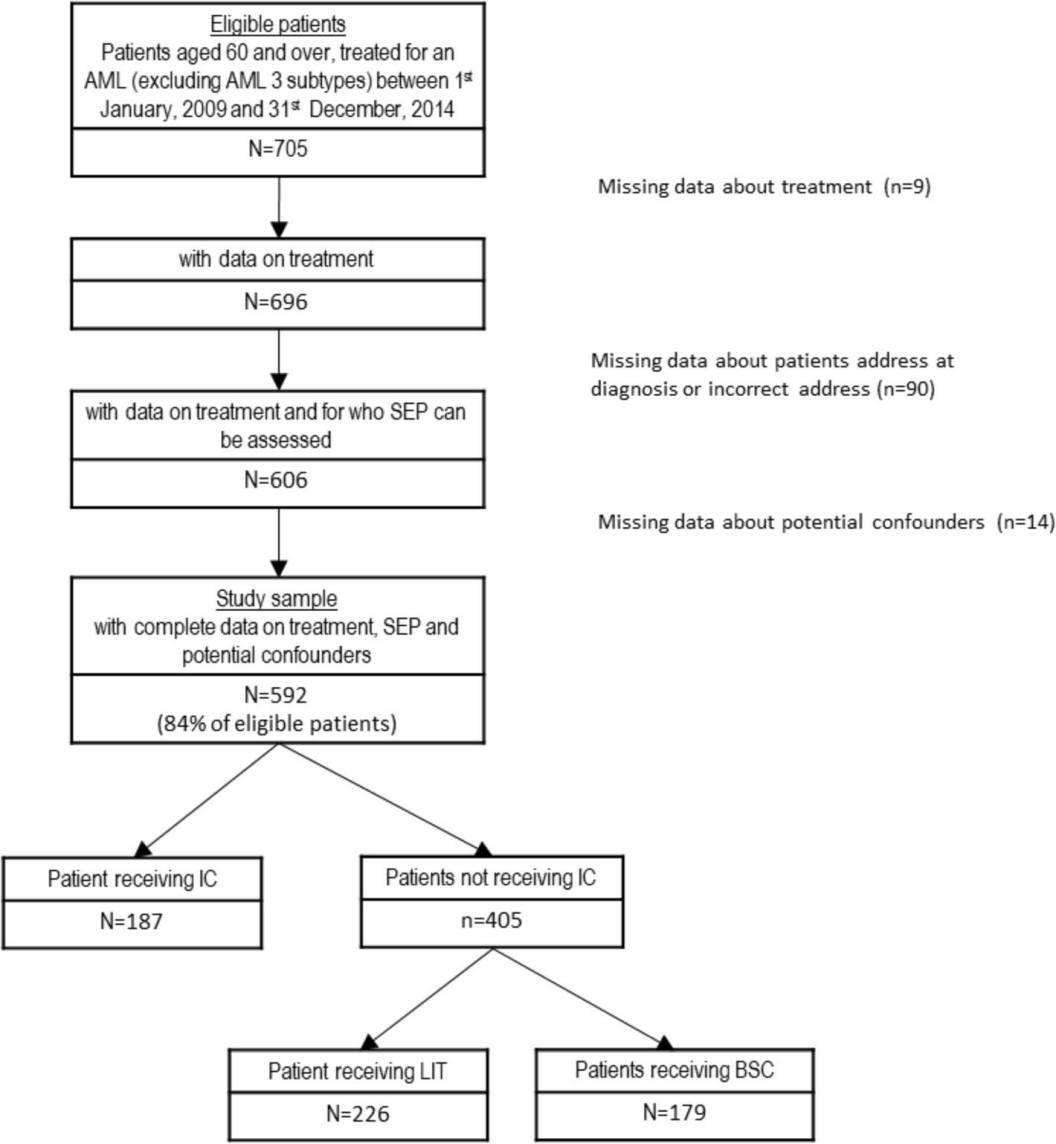
flowchart

The flowchart is presented in Fig. 1. Among the 705 eligible patients, 113 were excluded due to missing data on treatment, SEP, or covariates. The resulting study sample included 592 patients. As shown in Table 1, compared to these patients, those excluded were significantly older, less often men, more often treated by LIT (especially by low dose cytarabine), with less favorable clinical characteristics at the exception of white blood cell count for which no statistically significant difference was found, and their patients’ clinical characteristics were most often undefined. Excluded patients had also poorer overall survival (median survival [95%CI] in years = 0.18 [0.10; 0.42] versus 0.58 [0.45, 0.72] for included patients).

**Table 1:** Comparison between the excluded and the study samples characteristics (total *N* = 705)

		Excluded sample (*n* = 113)		Study sample (*n* = 592)		Test comparing study sample with excluded sample characteristics
		N	*% or mean (sd)*	N	*% or mean (sd)*	
Treatment (total *N* = 696)	Intensive chemotherapy	7	*7*	187	*32*	p* < 0.001
	Best supporting care	81	*12*	179	*30*	
	Hypomethylating agents	12	*3*	175	*7*	
	Aracytine low dose	3	*78*	42	*30*	
	Others	1	*1*	9	*1*	
Patient’s characteristics						
Age	Median (inter-quartile range)	80	*10*	74	*12*	*p* ^*#*^ *< 0.001*
Sex	Men	59	*52*	364	*61*	*p* ^*§*^ *= 0.065*
	Women	54	*48*	228	*39*	
Patients’ SEP (EDI quintile) (total *N* = 613)	Q1 – least deprived	3	*14*	124	*21*	
	Q2	6	*29*	104	*18*	
	Q3	6	*29*	127	*21*	p * = 0.388
	Q4	5	*24*	137	*23*	
	Q5 – most deprived	1	*5*	100	*17*	
Charlson comorbidity index	0	20	*19*	261	*44*	
	1	11	*10*	122	*21*	*p* ^*§*^ *= 0.001*
	2+	12	*11*	104	*18*	
	Undefinable	64	*60*	105	*18*	
Performance status	0/1	26	*25*	309	*52*	
	2	13	*13*	91	*15*	*p* ^*§*^ *= 0.001*
	3/4	10	*10*	60	*10*	
	Undefinable	55	*53*	132	*22*	
Tumor’s characteristics						
White blood cell counts (tercile) (total *N* = 599)	Tercile 1 – low	1	14	195	33	
	Tercile 2 – intermediate	4	57	189	32	p * = 0.393
	Tercile 3 – high	2	29	193	33	
	Undefinable	0	0	15	3	
AML ontogeny (total *N* = 704)	AML de novo	36	32	301	51	
	Secondary AML (post treatment / MDS)	41	37	268	45	*p* ^*§*^ *< 0.001*
	Undefinable	35	31	23	4	
Cytogenetic initial prognosis	Favorable/Intermediate	50	44	343	58	*p* ^*§*^ *< 0.001*
	Unfavorable	26	23	203	34	
	Undefinable	37	33	46	8	

*p*-value for Fisher test *, chi-square test §, or Wilcoxon #

### Description of the study population

From Table 1, IC, LIT and BSC represented respectively 32, 38 and 30% of the 592 patients included study sample. In total, 68% of the study sample (*n* = 405) did not receive IC. The distribution of patients between EDI levels was fairly balanced. Table 2 presented the distribution of patients’ characteristics according to their socioeconomic position. In bivariate analyses (Additional files 1, 2 and 3: Tables S1 to S3), poorer overall survival was associated with non-intensive therapy, the highest level of social deprivation, advanced age, higher level of comorbidity, poorer performance status, higher level of WBC, secondary or undefinable AML ontogeny, and unfavorable or undefinable cytogenetic prognosis (Additional file 1: Table S1). Regarding the treatment, using IC or non-IC was associated with social deprivation index, sex, age, comorbidity, performance status, WBC count, AML ontogeny, and cytogenetic prognosis (Additional file 2: Table S2). Among patients judged as not fit for IC, using low IT or BSC was associated with sex, comorbidity, performance status, and WBC count (Additional file 3: Table S3).

**Table 2:** Distribution of the study sample characteristics by patients’ socioeconomic position (*n* = 592)

		Patients’ SEP (EDI quintile)									
		0 (least deprived)		1		2		3		4 (most deprived)	
		n	%	n	%	n	%	n	%	n	%
Sex	Men	74	*59.68*	71	*68.27*	*70*	55.12	*86*	62.77	63	63.00
	Women	50	40.32	33	*31.73*	*57*	44.88	*51*	37.23	37	37.00
Age (median (interquartile range))		74	*15.5*	75	*13.00*	*74*	11.00	73	12.00	75	12.50
Charlson comorbidity index	0	59	47.58	*51*	49.04	54	42.52	61	44.53	36	36.00
	1	27	21.77	20	*19.23*	21	16.54	26	18.98	28	28.00
	2+	19	15.32	18	*17.31*	22	17.32	30	21.90	15	15.00
	Undefinable	19	15.32	*15*	*14.42*	30	23.62	20	14.60	21	21.00
Performance status	0/1	70	56.45	59	56.73	67	52.76	72	52.55	41	41.00
	2	14	11.29	18	17.31	15	11.81	23	16.79	21	21.00
	3/4	8	6.45	10	9.62	15	11.81	16	11.68	11	11.00
	Undefinable	32	25.81	17	16.35	30	23.62	26	*18.98*	27	27.00
White blood cell (terticle	Low	35	28.23	35	33.65	*37*	29.13	55	40.15	33	33.00
	Medium	43	34.68	36	34.62	*44*	34.65	38	27.74	28	28.00
	High	44	35.48	33	31.73	*41*	32.28	41	29.93	34	34.00
	Undefinable	2	1.61	0	0.00	*5*	3.94	3	2.19	5	5.00
AML ontogeny	AML de novo	74	59.68	51	49.04	63	49.61	73	53.28	40	40.00
	Secondary AML (post treatment / MDS)	46	37.10	49	47.12	59	46.46	60	43.80	54	54.00
	Undefinable	4	3.23	4	3.85	5	3.94	4	2.92	6	6.00
Cytogenetic prognosis	Favorable/Intermediate	77	62.10	54	51.92	76	59.84	84	61.31	52	52.00
	Unfavorable	38	30.65	44	42.31	39	30.71	42	30.66	40	40.00
	Undefinable	9	7.26	6	5.77	12	9.45	11	8.03	8	8.00
Treatment	Intensive chemotherapy	50	40.32	28	26.92	42	33.07	44	32.12	23	23.00
	Low intensive therapy	40	32.26	43	41.35	45	35.43	57	41.61	41	41.00
	Best supportive care	34	27.42	33	31.73	40	31.50	36	26.28	36	36.00

### Influence of SEP on overall survival

Table 3 presents the results from step 1 testing for the influence of patients’ SEP on overall survival. As shown by *model 1.0* results, compared to patients from the least deprived areas, those living in the most deprived areas had a higher risk of dying from all causes that was not explained by differences in age, sex or comorbidity. *Models 1.1 to 1.5* showed that the influence of the lowest SEP on survival was downsized to become not statistically significant after adjustment for AML ontogeny, and cytogenetic prognosis. Conversely, this effect resisted to adjustment for performance status, WBC and treatment. In *models 1.6* and *1.7*, we did not find any persisting influence of patients’ SEP on overall survival that was not explained by covariates presents in the models. Regarding the other factors, results from *models 1* showed that aging, poorer performance status levels, poor cytogenetic prognosis, and high values of WBC were associated with poorer survival. Results from the “time varying component” section indicates that the effect of WBC count on survival decreased with time from diagnosis.

**Table 3:** Step 1. Survival in association with patients’ SEP adjusted for treatment, patients’ and disease characteristics

Main components		Model 1.0 (M1.0)			Model 1.1			Model 1.2			Model 1.3			Model 1.4			Model 1.5			Model 1.6		M 1.7	
					M1.0 + perf. Status			M1.0 + AML ont			M1.0 + WBC.			M1.0 + cyto. Progn.			M1.0 + treatment			All but treatment		Fully adjusted	
		HR	[95% CI]		HR	[95% CI]		HR	[95% CI]		HR	[95% CI]		HR	[95% CI]		HR	[95% CI]		HR	[95% CI]	HR	[95% CI]
Age		1.04	[1.03; 1.05]		1.03	[1.02; 1.05]		1.04	[1.03; 1.05]		1.04	[1.03; 1.05]		1.04	[1.02; 1.05]		1.01	[1.00; 1.03]		1.03	[1.02; 1.05]	1.02	[1.00; 1.03]
Sex	Men	ref			ref			ref			ref						ref			Ref		ref	
	Women	0.88	[0.73; 1.07]		0.86	[0.71; 1.04]		0.87	[0.72; 1.05]		0.89	[0.74; 1.08]		0.84	[0.69; 1.01]		0.87	[0.72; 1.05]		0.81	[0.67; 0.98]	0.82	[0.68; 1.00]
Patients’ SEP (quintile of deprivation score)	Q1 – least	ref			Ref			Ref			Ref						Ref			ref		Ref	
	Q2	1.14	[0.85; 1.53]		1.11	[0.83; 1.50]		1.08	[0.80; 1.45]		1.17	[0.87; 1.35]		1.06	[0.79; 1.42]		1	[0.84; 1.50]		1.01	[0.75; 1.37]	0.96	[0.71; 1.29]
	Q3	0.89	[0.67; 1.18]		0.85	[0.64; 1.13]		0.83	[0.62; 1.11]		0.88	[0.66; 1.17]		0.89	[0.67; 1.18]		0.84	[0.69; 1.22]		0.80	[0.60; 1.07]	0.78	[0.58; 1.04]
	Q4	1.07	[0.82;	1.40]	1.02	[0.78; 1.33]		1.03	[0.79; 1.35]		1.07	[0.82; 1.40]		1.06	[0.81; 1.38]		1	[0.82; 1.40]		0.99	[0.75; 1.30]	0.94	[0.71; 1.24]
	Q5 – most	1.39	[1.04; 1.87]		1.37	[1.02; 1.84]		1.31	[0.97; 1.76]		1.47	[1.10; 1.97]		1.30	[0.97; 1.75]		1.35	[1.12; 2.01]		1.28	[0.95; 1.73]	1.29	[0.95; 1.74]
Charlson comorbidity index	0	ref			ref			ref			ref						ref			ref		ref	
	1	1.1	[0.86; 1.41]		1.02	[0.80; 1.31]		1.08	[0.85; 1.38]		1.15	[0.90; 1.47]		1.09	[0.86; 1.40]		1.01	[0.79; 1.29]		1.07	[0.83; 1.37]	1.00	[0.78; 1.28]
	2+	1.29	[1.01; 1.66]		1.18	[0.92; 1.52]		1.21	[0.94; 1.56]		1.3	[1.01; 1.66]		1.35	[1.06; 1.73]		1.13	[0.88; 1.45]		1.19	[0.92; 1.54]	1.08	[0.83; 1.41]
	Undefined	2.1	[1.60; 2.76]		1.89	[1.39; 2.56]		1.88	[1.40; 2.53]		2.06	[1.56; 2.73]		1.97	[1.49; 2.61]		1.29	[0.96; 1.74]		1.66	[1.20; 2.31]	1.25	[0.89; 1.75]
Performance status	0/1				ref															Ref		ref	
	2				1.51	[1.16; 1.97]														1.49	[1.15; 1.95]	1.50	[1.15; 1.95]
	3/4				2.36	[1.70; 3.28]														1.90	[1.34; 2.68]	1.72	[1.22; 2.42]
	Undefined				1.75	[1.24; 2.48]														1.52	[1.04; 2.20]	1.29	[0.88; 1.88]
AML ontogeny	AML de novo							ref												Ref		ref	
	Secondary AML (post treatment / MDS)							1.25	[1.03; 1.52]											1.21	[0.99; 1.48]	1.12	[0.91; 1.38]
	Undefined							1.56	[0.90; 2.72]											1.60	[0.90; 2.83]	1.52	[0.86; 2.70]
White blood cell (WBC) counts (tercile)	Tercile 1 – low										ref											Ref	
	Terticle 2 – intermediate										1.42	[1.10; 1.83]								1.38	[1.06; 1.79]	1.34	[1.04; 1.74]
	Terticle 3 – high										2.24	[1.66; 3.01]								2.16	[1.59; 2.93]	2.36	[1.74; 3.20]
	Undefined										2.9	[1.50; 5.60]								2.28	[1.14; 4.56]	2.10	[1.05; 4.21]
Cytogenetic prognosis	Favorable/Intermediate																						
	Unfavorable													2.00	[1.64; 2.43]					2.01	[1.64; 2.46]	1.72	[1.38; 2.13]
	Undefinable													1.99	[1.34; 2.96]					1.88	[1.24; 2.86]	1.44	[0.95; 2.18]
Treatment	Intensive chemotherapy																ref						
	Low intensive therapy																1.53	[1.19; 1.96]				1.36	[1.01; 1.82]
	Best supportive care																4.11	[2.93; 5.77]				3.24	[2.21; 4.76]
Time varying component																							
Time * Performance status					0.9998 [0.9997;0.9999]															0.9999 [0.9998; 1.0000]		0.9999 [0.9998; 1.0000]	
Time * White blood cell counts											0.9993 [0.9990; 0.9996]									0.9993 [0.9989; 0.9996]		0.9992 [0.9990; 0.9996]	

Adjusted hazard ratios [95% Confidence Intervals] of overall mortality from Adjusted Cox proportional hazards model with time dependent variables (*n* = 592)

### Influence of SEP on therapeutic strategies

Table 4 presents the results from step 2 testing for the influence of patients’ SEP on the probability of receiving or not IC. In *model 2.0* patients with the lowest SEP had lower access to IC than those with the highest SEP. From *the models 2.1* to 2*.4*, we observed that this association was downsized to become not statistically significant after adjustment for AML ontogeny, and cytogenetic prognosis but it was not affected by adjustment for performance status and WBC count. In *model 2.5* results, patients’ SEP had no more influence on the use of IC. Regarding the other factors, *model 2.5* shows that the probability of receiving IC was lower among older patients, undefinable comorbidity level, poorer performance status, secondary (post-treatment or MDS) AML, and unfavorable cytogenetic prognosis. Conversely, higher level of white blood cell count was associated with higher probability of receiving IC.

**Table 4:** Step 2. Models of the association linking patients’ SEP to receiving IC

		Model 2.0 (M2.0)			Model 2.1		Model 2.2		Model 2.3			Model 2.4			Model 2.5	
					M2.0 + perf. Status		M2.0 + AML ontogeny		M2.0 + WBC			M2.0 + cytogen. Progn.			Fully adjusted	
		OR	[95% CI]		OR	[95% CI]	OR	[95% CI]	OR	[95% CI]		OR	[95% CI]		OR	[95% CI]
Age		0.79	[0.76; 0.82]		0.79	[0.76; 0.82]	0.76	[0.73; 0.80]	0.77	[0.74; 0.81]		0.76	[0.73; 0.80]		0.74	[0.70; 0.79]
Sex	Men	ref			ref		ref		ref						ref	
	Women	0.65	[0.40; 1.05]		0.65	[0.40; 1.04]	0.61	[0.36; 1.03]	0.73	[0.44; 1.22]		0.84	[0.50; 1.44]		0.71	[0.40; 1.24]
Patients’ SEP (quintile of deprivation score)	Q1 – least	ref			ref		Ref		ref						ref	
	Q2	0.46	[0.22; 0.99]		0.46	[0.21;0.99]	0.52	[0.23; 1.17]	0.43	[0.20; 0.96]		0.47	[0.20; 1.09]		0.47	[0.20; 1.14]
	Q3	0.98	[0.49; 1.95]		0.97	[0.48; 1.95]	1.07	[0.51; 2.25]	0.97	[0.47; 2.01]		0.77	[0.36; 1.65]		1.08	[0.48; 2.41]
	Q4	0.55	[0.28; 1.08]		0.56	[0.28; 1.12]	0.58	[0.28; 1.20]	0.54	[0.27; 1.09]		0.45	[0.21; 0.96]		0.58	[0.26; 1.27]
	Q5 – most	0.41	[0.19; 0.90]		0.45	[0.20; 0.98]	0.59	[0.25; 1.40]	0.40	[0.17; 0.93]		0.45	[0.19; 1.07]		0.60	[0.23; 1.53]
Charlson comorbidity index	0	ref			ref		ref		ref						ref	
	1	0.61	[0.33; 1.13]		0.63	[0.34; 1.17]	0.66	[0.34; 1.29]	0.58	[0.30; 1.10]		0.70	[0.36; 1.37]		0.69	[0.34; 1.40]
	2+	0.64	[0.35; 1.17]		0.66	[0.35; 1.22]	1.05	[0.54; 2.03]	0.55	[0.29; 1.05]		0.59	[0.30; 1.15]		1.06	[0.52; 2.18]
	Undefinable	0.14	[0.06; 0.35]		0.18	[0.07; 0.46]	0.19	[0.07; 0.50]	0.13	[0.05; 0.32]		0.31	[0.12; 0.82]		0.18	[0.06; 0.55]
Performance status	0/1				ref										Ref	
	2				0.45	[0.23; 0.86]									0.38	[0.18; 0.81]
	3/4				0.77	[0.34; 1.74]									0.3	[0.11; 0.82]
	Undefinable				0.6	[0.27; 1.36]									0.74	[0.30; 1.83]
AML ontogeny	AML de novo						ref								ref	
	Secondary (post MDS or post treatment)						0.13	[0.07; 0.23]							0.12	[0.06; 0.22]
	Undefinable						Not estimated								Not estimated	
White blood cell (WBS) counts (tercile)	Tercile 1 – low								Ref						ref	
	Tercile 2 – intermediate								1.5	[0.82; 2.75]					1.86	[0.95; 3.65]
	Tercile 3 – high								5.74	[3.06; 10.79]					7.83	[3.82; 16.08]
	Undefinable ^a^								Not estimated						Not estimated	
Cytogenetic prognosis	Favorable/Intermediate											ref			ref	
	Unfavorable											0.14	[0.08; 0.25]		0.12	[0.06; 0.22]
	Undefinable ^a^											Not estimated			Not estimated	

Models are adjusted for each confounder, and fully adjusted. Generalized linear model with logit link function, adjusted odds ratios [95% Confidence Intervals] (*N* = 592)

^a^the perfect predictor of outcome “undefinable” AML ontogeny, White blood cell counts, and cytogenetic prognosis were retained in the models to avoid reducing sample size, but OR and 95%CI were not estimated

Table 5 presents the results from step 3 testing for the influence of deprivation on the probability of receiving low intensive therapy or not, i.e. BSC, among patients judged unfit for IC (*n* = 405). Results from models 3.0 to 3.3 did not show any statistically significant influence of patients’ SEP. Regarding the other factors, as expected, ageing, comorbidity, poorer performance status levels, and higher WBC count were associated with lower probability of receiving LIT.

**Table 5:** Step 3. Adjusted models of the association linking patients’ SEP to receiving non-intensive therapy (*n* = 405)

		Model 3.0 (M3.0)		Model 3.1		Model 3.2		Model 3.3	
				M3.0 + perf. Status		M3.0 + WBC		Fully adjusted	
		OR	[95% CI]	OR	[95% CI]	OR	[95% CI]	OR	[95% CI]
Age		0.94	[0.91; 0.97]	0.96	[0.92; 0.99]	0.95	[0.92; 0.98]	0.96	[0.93; 0.99]
Sex	Men	ref		ref		ref		ref	
	Women	1.39	[0.88; 2.20]	1.56	[0.96; 2.52]	1.41	[0.88; 2.25]	1.56	[0.96; 2.55]
Patients’ SEP (quintile of deprivation score)	Q1 – least	ref		ref		ref		ref	
	Q2	1.02	[0.50; 2.09]	0.89	[0.42; 1.89]	0.94	[0.45; 1.98]	0.85	[0.40; 1.84]
	Q3	1.09	[0.54; 2.20]	1.05	[0.50; 2.21]	1.07	[0.52; 2.19]	1.06	[0.50; 2.26]
	Q4	1.12	[0.56; 2.25]	1.04	[0.51; 2.13]	1.04	[0.51; 2.12]	1.00	[0.48; 2.08]
	Q5 – most	1.05	[0.51; 2.17]	1.05	[0.50; 2.22]	1.06	[0.50; 2.24]	1.07	[0.50; 2.32]
Charlson comorbidity index	0	ref		ref		ref		ref	
	1	0.42	[0.23; 0.75]	0.47	[0.26; 0.86]	0.43	[0.23; 0.77]	0.47	[0.25; 0.86]
	2+	0.43	[0.23; 0.80]	0.51	[0.27; 0.98]	0.42	[0.22; 0.79]	0.49	[0.25; 0.94]
	Undefinable	0.10	[0.06; 0.20]	0.15	[0.07; 0.29]	0.12	[0.06; 0.22]	0.15	[0.08; 0.31]
Performance status	0/1			ref				ref	
	2			0.88	[0.46; 1.68]			0.92	[0.48; 1.79]
	3/4			0.21	[0.10; 0.45]			0.24	[0.11; 0.52]
	Undefinable			0.36	[0.20; 0.65]			0.41	[0.22; 0.76]
White blood cell (WBS) counts (tercile)	Tercile 1 – low					ref		ref	
	Tercile 2 – intermediate					0.76	[0.44; 1.30]	0.85	[0.49; 1.49]
	Tercile 3 – high					0.41	[0.23; 0.72]	0.51	[0.28; 0.92]
	Undefinable					0.13	[0.03; 0.71]	0.16	[0.03; 0.93]

Population is selected among those who were not considered for intensive chemotherapy. Generalized linear model with logit link function, adjusted odds ratios [95% Confidence Intervals]

### Sensitivity analyses

In sensitivity analyses, we found the same pattern of results but with larger confidence intervals. The detailed results are presented in Additional files 4, 5 and 6: Tables S4, S5 and S6.

## Discussion

We found an association linking patients’ SEP to overall survival that did not persist after adjustment for AML and patients’ characteristics. As expected, the type of treatment was strongly associated with survival. However, its role as intermediate factor in the pathway linking patients’ SEP to survival is not supported by our results. Indeed, we showed a statistically significant lower propensity of being treated using intensive chemotherapy among patients with lowest SEP but this did not persist after adjustment for AML ontogeny and cytogenetic prognosis. This may indicate that, patients’ and AML initial characteristics being equal, patients’ SEP do not influence the utilization of intensive chemotherapy. However, we cannot exclude an indirect influence of patients’ SEP on the utilization of intensive chemotherapy and survival through SEP-related differences in AML initial presentation and cytogenetic prognosis. No such influence of patients’ SEP was found on the propension of having low intensive therapy or BSC among patients judged unfit for IC.

This study aimed at testing for SEP-related differences in cancer management and outcome among old patients (60 years and over) in a setting of a national tax-supported healthcare system. We used data from an ongoing prospective observational cohort including all patients newly diagnosed for an AML in the South-West of France since 2007. In France, the healthcare organization is centralized and relayed at the regional level by Regional Health Agency. Many efforts were done for standardizing and harmonizing cancer management, notably with the implementation of the national cancer plans which aimed, amongst others, at developing regional cancer coordination centers responsible of the holding of multidisciplinary team meeting (MTM) for the first plan (2003–2007) and the reduction of social and territorial inequalities in cancer management for the second and third plans (2009–2013/2014–2019). One role of the regional cancer coordination centers is notably to ensure the diffusion of clinical guidelines throughout all the region centers. Thus, despite the lack of data for the whole national territory, we assumed that it is unlikely to affect the generalization of our results. However, our results showed that patients excluded from the study were not different regarding SEP but had less often intensive treatment, less favorable clinical characteristics and poorer survival. Thus, we may have underestimated the influence of SEP on both treatment and survival. Lastly, data were collected from medical files which did not contain any information on individual SEP like patients’ occupation or education level or income. Therefore, we used an ecological deprivation index to approach individual SEP despite the exposure to potential ecological fallacy. Indeed, as we attributed to patients the deprivation level of their living area to approach their individual SEP, it is possible that this measure hides some contextual dimension, like for instance environmental exposures. However, this is lessened as we used the French European Deprivation Index (EDI) at the smallest geographical area (the IRIS corresponding to approximately 2000 individuals) for which census data of the French population are available. The EDI has been previously used as patients’ individual SEP proxy in studies dealing with social inequalities in cancer incidence [26], management [27] and outcome [28]. Moreover, a study published in early 2017 compared several deprivation indexes including the European Deprivation index (EDI), all aggregated at the IRIS level, and showed that the EDI was quite good “proxies” for individual deprivation (Area Under the Curve close to 0.7) [29].

To our knowledge, we found only two studies addressing SEP-related differences in AML management or outcome in a tax-supported healthcare setting. Regarding survival, our results cannot be compared to Kristinsson et al.’s [14] which concerns all AML patients without age restriction. In addition, we cannot compare our results to Østgård et al.’s study as they assessed SEP influence on survival only among patients selected for intensive chemotherapy [15]. In our study, we did not find any independent effect of patients’ SEP after adjustment for both patients’ and tumor’s characteristics among patients aged of at least 60 years. More specifically, we found a SEP influence on survival that persisted in model adjusted for performance status, and WBC. This influence was reduced after adjustment for treatment and was downsized to become no more significant with adjustment for AML ontogeny, and cytogenetic prognosis. This suggested an indirect influence of SEP on survival through initial SEP-related differences in AML presentation even if we could not exclude, regarding to the slightly attenuation of the effect size, that the insignificant effect was due to lack of statistical power. When we consider the treatment utilization, the focus on tax-supported healthcare setting limits theoretically the effect of financial barrier to access to care. Østgård and colleagues’ study supported the association between access to intensive therapy and education, as a proxy of SEP, among all patients as well as patients older than 60. In addition, they found an independent effect of education after controlling for occupation, marital status and income on intensive treatment among older patients. No associations with income were found [15]. In our study, we found a lower access to intensive therapy among patients with the lowest SEP which persisted in model adjusted for performance status and WBC count but was downsized to become no more significant when accounting for AML ontogeny, and cytogenetic. This reinforced the role of the AML initial presentation in the SEP-survival association discussed above. Among patients who were judged unfit for intensive therapy, we found no more influence of patients’ SEP. Finally compared to Østgård and colleagues’ study, we did not show any independent persisting influence of SEP on survival and treatment allocation. This may indicate that, in our study region, patients’ and AML initial characteristics being equal, patients’ SEP do not influence the way AML is treated nor its outcome. An indirect influence of patients’ SEP on the utilization of intensive chemotherapy and survival is more likely through SEP-related differences in AML initial presentation and cytogenetic prognosis. Compared to Østgård and colleagues’ study, the absence of persisting influence of SEP in our study may derive, at least partially, from differences in the study design as their study was based on populational registry whereas ours included patients from their entrance into the healthcare system. However, this also illustrates the variability of the mechanisms linking patients’ SEP to survival trough, for instance, differences in management or in initial presentation depending potentially to various SEP dimensions.

## Conclusions

The hypothesis of an indirect influence of SEP on survival through SEP-related differences in treatment utilization is not supported by our results, at least for the initial treatment. Adjusting survival model for treatment did not neutralize the SEP influence which seems rather to derive from SEP-related difference in AML ontogeny and cytogenetic prognosis. It therefore appears necessary to continue the investigation beyond the limits of treatment initiation and survival to identify at which points in the course of treatment, factors that might be considered as clinically irrelevant may be involved in the patient care trajectory. Especially further analyses are needed to test formally the assumption of an indirect influence of patients’ SEP on survival through AML initial presentation and cytogenetic prognosis.

## Additional files

Additional file 1: **Table S1.** Bivariate associations between covariates and overall survival. (DOCX 17 kb)

Additional file 2: **Table S2.** Bivariate associations between covariates and treatment selection in terms of intensive chemotherapy or not. (DOCX 15 kb)

Additional file 3: **Table S3.** Bivariate associations between covariates and treatment selection in terms of Low intensive chemotherapy or BSC. (DOCX 15 kb)

Additional file 4: **Table S4.** Step 1 sensitivity analysis. Survival in association with patients’ SEP adjusted for treatment, patients’ and disease characteristics. Adjusted hazard ratios [95% Confidence Intervals] of overall mortality from Adjusted Cox proportional hazards model with time dependent variables (*n* = 684). (DOCX 23 kb)

Additional file 5: **Table S5.** Step 2 Sensitivity analysis. Models of the association linking patients’ SEP to receiving Intensive Chemotherapy adjusted for each confounder, and fully adjusted. Generalized linear model with logit link function, adjusted odds ratios [95% Confidence Intervals], with missing data treated by multiple imputation (*n* = 685). (DOCX 20 kb)

Additional file 6: **Table S6.** Step 3 Sensitivity analysis. Adjusted models of the association linking patients’ SEP to receiving non-intensive therapy among those who were not considered for intensive chemotherapy (*n* = 498). Generalized linear model with logit link function, adjusted odds ratios [95% Confidence Intervals] after treating missing data using multiple imputation. (DOCX 17 kb)

## Data Availability

The datasets used and/or analysed during the current study are available from the corresponding author on reasonable request.

## Abbreviations

AML: Acute myeloid leukemia
BSC: Best supportive care
EDI: European deprivation index
IC: Intensive chemotherapy
LIT: Low intensive therapy
SEP: Socioeconomic position
