# Supplementary material for: Are social inequalities in acute myeloid leukemia survival explained by differences in treatment utilization? Results from a French longitudinal observational study among older patients
Source: BMC Cancer. 2019 Sep 5;19:883. doi: 10.1186/s12885-019-6093-3 (PMC6729078; doi:10.1186/s12885-019-6093-3)
Supplement: Supplementary file 1 — Table S1. Bivariate associations between covariates and overall survival. (DOCX 17 kb) [file 12885_2019_6093_MOESM1_ESM.docx]

Table S1: Bivariate associations between covariates and overall survival

|  |  | **not dead** | | **dead** | | **cox proportional hazard bivariate models** | | | | |
| --- | --- | --- | --- | --- | --- | --- | --- | --- | --- | --- |
|  |  | N (%) | | N (%) | | **HR** | **p-value** | **[95%** | **CI]** | **global p-value** |
| **Treatment (n=696)** | CI | 44 | *40* | 143 | *30* | 1.72 | 0.000 | 1.39 | 2.14 | 0.000 |
|  | LIT | 17 | *15* | 209 | *43* | 5.22 | 0.000 | 4.03 | 6.77 |  |
|  | BSC | 49 | *45* | 130 | *27* |  |  |  |  |  |
| **Patient's characteristics** | | | | | | | | | | |
| **Quintile EDI (n=613)** | 0 | 24 | *22* | 100 | *21* |  |  |  |  |  |
|  | 1 | 21 | *19* | 83 | *17* | 1.11 | 0.483 | 0.83 | 1.49 | 0.060 |
|  | 2 | 32 | *29* | 95 | *20* | 0.96 | 0.792 | 0.73 | 1.28 |  |
|  | 3 | 17 | *15* | 120 | *25* | 1.08 | 0.578 | 0.83 | 1.41 |  |
|  | 4 | 16 | *15* | 84 | *17* | 1.45 | 0.012 | 1.09 | 1.94 |  |
| **Sex** | Men | 58 | *53* | 306 | *63* |  |  |  |  |  |
|  | Women | 52 | *47* | 176 | *37* | 0.88 | 0.185 | 0.73 | 1.06 |  |
| **Age (mean (sd))** |  | 74 | *9* | 74 | *8* | 1.05 | 0.000 | 1.04 | 1.06 |  |
| **Charlson comorbidity index** | 0 | 48 | *44* | 213 | *44* |  |  |  |  |  |
|  | 1 | 25 | *23* | 97 | *20* | 1.24 | 0.079 | 0.98 | 1.58 | 0.000 |
|  | 2+ | 13 | *12* | 91 | *19* | 1.33 | 0.022 | 1.04 | 1.70 |  |
|  | Undefinable | 24 | *22* | 81 | *17* | 2.59 | 0.000 | 2.00 | 3.36 |  |
| **Performance status** | 0/1 | 63 | *57* | 246 | *51* |  |  |  |  |  |
|  | 2 | 10 | *9* | 81 | *17* | 1.62 | 0.000 | 1.26 | 2.09 | 0.000 |
|  | 3/4 | 10 | *9* | 50 | *10* | 2.21 | 0.000 | 1.63 | 3.00 |  |
|  | Undefinable | 27 | *25* | 105 | *22* | 2.16 | 0.000 | 1.72 | 2.72 |  |
| **Tumor's characteristics** | | | | | | | | | | |
| **White blood cell (tercile) (n=599))** | Low | 37 | 34 | 158 | 33 |  |  |  |  |  |
|  | Medium | 36 | 33 | 153 | 32 | 1.08 | 0.495 | 0.86 | 1.35 | 0.000 |
|  | High | 33 | 30 | 160 | 33 | 1.26 | 0.038 | 1.01 | 1.58 |  |
|  | Undefinable | 4 | 4 | 11 | 2 | 3.43 | 0.000 | 1.85 | 6.35 |  |
| **Initial status (n=704)** | De novo | 61 | 55 | 240 | 50 |  |  |  |  |  |
|  | Secondary (post MDS or post treatment) | 43 | 39 | 225 | 47 | 1.37 | 0.001 | 1.15 | 1.65 | 0.001 |
|  | Undefinable | 6 | 5 | 17 | 4 | 3.86 | 0.000 | 2.34 | 6.35 |  |
| **Cytogenetic prognosis** | Favorable/Intermediate | 80 | 73 | 263 | 55 |  |  |  |  |  |
|  | Unfavorable | 19 | 17 | 184 | 38 | 2.00 | 0.000 | 1.64 | 2.42 | 0.000 |
|  | Undefinable | 11 | 10 | 35 | 7 | 3.79 | 0.000 | 2.65 | 5.43 |  |
