# Supplementary material for: Are social inequalities in acute myeloid leukemia survival explained by differences in treatment utilization? Results from a French longitudinal observational study among older patients
Source: BMC Cancer. 2019 Sep 5;19:883. doi: 10.1186/s12885-019-6093-3 (PMC6729078; doi:10.1186/s12885-019-6093-3)
Supplement: Supplementary file 2 — Table S2. Bivariate associations between covariates and treatment selection in terms of intensive chemotherapy or not. (DOCX 15 kb) [file 12885_2019_6093_MOESM2_ESM.docx]

Table S2: Bivariate associations between covariates and treatment selection in terms of intensive chemotherapy or not.

| n=592 |  | **non-IC** | | **IC** | |  |
| --- | --- | --- | --- | --- | --- | --- |
|  |  | N (*%* ) | | N (% ) | | ***p-value **** |
| **Patient's characteristics** | | | | | | |
| Patients’ SEP (quintile of deprivation score) | Q1 – least | 74 | *18* | 50 | *27* |  |
|  | Q2 | 76 | *19* | 28 | *15* | *0.061* |
|  | Q3 | 85 | *21* | 42 | *22* |  |
|  | Q4 | 93 | *23* | 44 | *24* |  |
|  | Q5 – most | 77 | *19* | 23 | *12* |  |
| Sex | Men | 240 | *59* | 124 | *66* | *0.101* |
|  | Women | 165 | *41* | 63 | *34* |  |
| Age |  | 77 | *7* | 67 | *5* | *0.000* |
| Charlson comorbidity index | 0 | 149 | *37* | 112 | *60* |  |
|  | 1 | 87 | *21* | 35 | *19* | *0.007* |
|  | 2+ | 73 | *18* | 31 | *17* |  |
|  | Undefinable | 96 | *24* | 9 | *5* |  |
| Performance status | 0/1 | 176 | *43* | 133 | *71* |  |
|  | 2 | 68 | *17* | 23 | *12* | *0.001* |
|  | 3/4 | 45 | *11* | 15 | *8* |  |
|  | Undefinable | 116 | *29* | 16 | *9* |  |
| **Tumor's characteristics** | | | | | | |
| White blood cell (tercile) | Low | 152 | 38 | 43 | 23 |  |
|  | Medium | 132 | 33 | 57 | 30 | *<0.001* |
|  | High | 106 | 26 | 87 | 47 |  |
|  | Undefinable | 15 | 4 | 0 | 0 |  |
| Initial status | De novo | 163 | 40 | 138 | 74 |  |
|  | post-affection | 219 | 54 | 49 | 26 | *<0.001* |
|  | Undefinable | 23 | 6 | 0 | 0 |  |
| Cytogenetic prognosis | Favorable/Intermediate | 192 | 47 | 151 | 81 |  |
|  | Unfavorable | 167 | 41 | 36 | 19 | *<0.001* |
|  | Undefinable | 46 | 11 | 0 | 0 |  |
| *** chi2 or student test** | |  |  |  |  |  |
