# Supplementary material for: Are social inequalities in acute myeloid leukemia survival explained by differences in treatment utilization? Results from a French longitudinal observational study among older patients
Source: BMC Cancer. 2019 Sep 5;19:883. doi: 10.1186/s12885-019-6093-3 (PMC6729078; doi:10.1186/s12885-019-6093-3)
Supplement: Supplementary file 3 — Table S3. Bivariate associations between covariates and treatment selection in terms of Low intensive chemotherapy or BSC. (DOCX 15 kb) [file 12885_2019_6093_MOESM3_ESM.docx]

Table S3: Bivariate associations between covariates and treatment selection in terms of Low intensive chemotherapy or BSC

| **n=405** |  | **BSC** | | **LIT** | |  |
| --- | --- | --- | --- | --- | --- | --- |
|  |  | **N *(%)*** | | **N *(%)*** | | ***p value **** |
| **Patient's characteristics** | | | | | | |
| **Quintile EDI (n=613)** | **0** | 34 | *19* | 40 | *18* | *0.785* |
|  | **1** | 33 | *18* | 43 | *19* |  |
|  | **2** | 40 | *22* | 45 | *20* |  |
|  | **3** | 36 | *20* | 57 | *25* |  |
|  | **4** | 36 | *20* | 41 | *18* |  |
| **Sex** | **Men** | 113 | *63* | 127 | *56* | *0.158* |
|  | **Women** | 66 | *37* | 99 | *44* |  |
| **Age (mean (sd))** | | 79 | *8* | 76 | *6* |  |
| **Charlson comorbidity index** | **0** | 35 | *20* | 114 | *50* |  |
|  | **1** | 39 | *22* | 48 | *21* |  |
|  | **2+** | 32 | *18* | 41 | *18* | *0.001* |
|  | **Undefinable** | 73 | *41* | 23 | *10* |  |
| **Performance status** | **0/1** | 48 | *27* | 128 | *57* |  |
|  | **2** | 21 | *12* | 47 | *21* | *0.000* |
|  | **3/4** | 29 | *16* | 16 | *7* |  |
|  | **Undefinable** | 81 | *45* | 35 | *15* |  |
| **Tumor's characteristics** | | | | | | |
| **White blood cell (tercile (n=599))** | **Low** | 50 | 28 | 102 | 45 |  |
|  | **Medium** | 53 | 30 | 79 | 35 | <0.001 |
|  | **High** | 63 | 35 | 43 | 19 |  |
|  | **Undefinable** | 13 | 7 | 2 | 1 |  |
| **Initial status (n=704)** | **De novo** | 66 | 37 | 97 | 43 |  |
|  | **post-affection** | 93 | 52 | 126 | 56 | 0.699 |
|  | **Undefinable** | 20 | 11 | 3 | 1 |  |
| **Cytogenetic prognosis** | **Favorable/Intermediate** | 73 | 41 | 119 | 53 |  |
|  | **Unfavorable** | 66 | 37 | 101 | 44 | 0.771 |
|  | **Undefinable** | 40 | 22 | 6 | 3 |  |
| *** chi2 or student test** | |  |  |  |  |  |
