# Supplementary material for: Are social inequalities in acute myeloid leukemia survival explained by differences in treatment utilization? Results from a French longitudinal observational study among older patients
Source: BMC Cancer. 2019 Sep 5;19:883. doi: 10.1186/s12885-019-6093-3 (PMC6729078; doi:10.1186/s12885-019-6093-3)
Supplement: Supplementary file 4 — Table S4. Step 1 sensitivity analysis. Survival in association with patients’ SEP adjusted for treatment, patients’ and disease characteristics. Adjusted hazard ratios [95% Confidence Intervals] of overall mortality from Adjusted Cox proportional hazards model with time dependent variables (n = 684). (DOCX 23 kb) [file 12885_2019_6093_MOESM4_ESM.docx]

Table S4: Step 1 sensitivity analysis. Survival in association with patients’ SEP adjusted for treatment, patients’ and disease characteristics. Adjusted hazard ratios [95% Confidence Intervals] of overall mortality from Adjusted Cox proportional hazards model with time dependent variables (n=684).

| n=684 | | Model 5.0 (M3.0) | | | Model 5.1 | | | Model 5.2 | | | Model 5.3 | | | Model 5.4 | | | Model 5.5 | | | Model 5.6 | | | Model 5.7 | | |
| --- | --- | --- | --- | --- | --- | --- | --- | --- | --- | --- | --- | --- | --- | --- | --- | --- | --- | --- | --- | --- | --- | --- | --- | --- | --- |
|  |  |  |  |  | M5.0 + perf. status | | | M5.0 + AML ont | | | M5.0 + WBC. | | | M5.0 + cyto. Progn. | | | M5.0 + treatment | | | All but treatment | | | Fully adjusted | | |
| **Main components** | | HR | [95% | CI] | HR | [95% | CI] | HR | [95% | CI] | HR | [95% | CI] | HR | [95% | CI] | HR | [95% | CI] | HR | [95% | CI] | HR | [95% | CI] |
| Age | | 1.04 | [1.03; | 1.05] | 1.03 | [1.02; | 1.04] | 1.04 | [1.03; | 1.05] | 1.04 | [1.03; | 1.05] | 1.03 | [1.02; | 1.05] | 1.01 | [1.00; | 1.02] | 1.03 | [1.02; | 1.04] | 1.01 | [1.00; | 1.03] |
| Sex | Men | ref |  |  | ref |  |  | ref |  |  | ref |  |  |  |  |  | ref |  |  | Ref |  |  | ref |  |  |
|  | Women | 0.89 | [0.75; | 1.07] | 0.87 | [0.73; | 1.04] | 0.88 | [0.73; | 1.05] | 0.90 | [0.75; | 0.84] | 0.70 | [1.00; | 1.01] | 0.86 | [0.72; | 1.03] | 0.81 | [0.68; | 0.97] | 0.81 | [0.68; | 0.98] |
| Patients’ SEP (quintile of deprivation score) | Q1 – least | ref |  |  | Ref |  |  | Ref |  |  | Ref |  |  |  |  |  | Ref |  |  | ref |  |  | Ref |  |  |
|  | Q2 | 1.13 | [0.85; | 1.50] | 1.11 | [0.83; | 1.48] | 1.08 | [0.81; | 1.44] | 1.15 | [0.86; | 1.53] | 1.06 | [0.79; | 1.41] | 0.99 | [0.74; | 1.33] | 1.02 | [0.76; | 1.37] | 0.96 | [0.71; | 1.29] |
|  | Q3 | 0.89 | [0.68; | 1.17] | 0.86 | [0.65; | 1.13] | 0.85 | [0.64; | 1.12] | 0.89 | [0.68; | 1.16] | 0.89 | [0.68; | 1.18] | 0.86 | [0.65; | 1.13] | 0.82 | [0.62; | 1.09] | 0.81 | [0.61; | 1.09] |
|  | Q4 | 1.05 | [0.81; | 1.37] | 1.01 | [0.78; | 1.31] | 1.02 | [0.79; | 1.33] | 1.06 | [0.82; | 1.37] | 1.04 | [0.80; | 1.36] | 0.99 | [0.76; | 1.28] | 0.98 | [0.75; | 1.28] | 0.93 | [0.71; | 1.23] |
|  | Q5 – most | 1.34 | [1.00; | 1.79] | 1.32 | [0.98; | 1.77] | 1.27 | [0.94; | 1.70] | 1.40 | [1.04; | 1.87] | 1.27 | [0.94; | 1.70] | 1.30 | [0.96; | 1.76] | 1.25 | [0.92; | 1.70] | 1.27 | [0.92; | 1.74] |
| Charlson comorbidity index | 0 | ref |  |  | ref |  |  | ref |  |  | ref |  |  |  |  |  | ref |  |  | ref |  |  | ref |  |  |
|  | 1 | 1.12 | [0.89; | 1.43] | 1.04 | [0.82; | 1.32] | 1.11 | [0.87; | 1.41] | 1.16 | [0.92; | 1.48] | 1.11 | [0.87; | 1.41] | 1.03 | [0.81; | 1.31] | 1.08 | [0.85; | 1.38] | 1.01 | [0.79; | 1.29] |
|  | 2+ | 1.29 | [1.01; | 1.63] | 1.18 | [0.93; | 1.50] | 1.23 | [0.96; | 1.57] | 1.29 | [1.02; | 1.64] | 1.37 | [1.08; | 1.74] | 1.11 | [0.87; | 1.41] | 1.22 | [0.96; | 1.56] | 1.11 | [0.86; | 1.42] |
|  | Undefined | 2.28 | [1.78; | 2.92] | 2.00 | [1.52; | 2.63] | 2.06 | [1.58; | 2.68] | 2.21 | [1.72; | 2.85] | 2.11 | [1.64; | 2.73] | 1.39 | [1.06; | 1.83] | 1.77 | [1.32; | 2.39] | 1.34 | [0.99; | 1.83] |
| Performance status | 0/1 |  |  |  | ref |  |  |  |  |  |  |  |  |  |  |  |  |  |  | Ref |  |  | ref |  |  |
|  | 2 |  |  |  | 1.54 | [1.19; | 1.99] |  |  |  |  |  |  |  |  |  |  |  |  | 1.52 | [1.18; | 1.97] | 1.44 | [1.11; | 1.86] |
|  | 3/4 |  |  |  | 2.50 | [1.83; | 3.41] |  |  |  |  |  |  |  |  |  |  |  |  | 2.05 | [1.48; | 2.85] | 1.69 | [1.22; | 2.35] |
|  | Undefined |  |  |  | 1.88 | [1.36; | 2.59] |  |  |  |  |  |  |  |  |  |  |  |  | 1.61 | [1.14; | 2.28] | 1.20 | [0.90; | 1.61] |
| AML ontogeny | AML de novo |  |  |  |  |  |  | ref |  |  |  |  |  |  |  |  |  |  |  | Ref |  |  | ref |  |  |
|  | Secondary AML (post treatment / MDS) | | | | |  |  | 1.22 | [1.01; | 1.46] |  |  |  |  |  |  |  |  |  | 1.18 | [0.98; | 1.43] | 1.07 | [0.87; | 1.30] |
|  | Undefined |  |  |  |  |  |  | 1.53 | [0.96; | 2.43] |  |  |  |  |  |  |  |  |  | 1.47 | [0.90; | 2.40] | 1.35 | [0.82; | 2.22] |
| White blood cell (WBC) counts (tercile) | Tercile 1 – low |  |  |  |  |  |  |  |  |  | ref |  |  |  |  |  |  |  |  |  |  |  | Ref |  |  |
|  | Terticle 2 – intermediate | | | |  |  |  |  |  |  | 1.35 | [1.04; | 1.77] |  |  |  |  |  |  | 1.29 | [0.99; | 1.69] | 1.26 | [0.96; | 1.65] |
|  | Terticle 3 – high | | | |  |  |  |  |  |  | 2.06 | [1.51; | 2.80] |  |  |  |  |  |  | 1.96 | [1.43; | 2.68] | 2.13 | [1.55; | 2.93] |
|  | Undefined |  |  |  |  |  |  |  |  |  | 2.55 | [1.33; | 4.88] |  |  |  |  |  |  | 2.04 | [1.02; | 4.11] | 1.98 | [0.96; | 4.10] |
| Cytogenetic prognosis | Favorable/Intermediate | |  |  |  |  |  |  |  |  |  |  |  |  |  |  |  |  |  |  |  |  |  |  |  |
|  | Unfavorable |  |  |  |  |  |  |  |  |  |  |  |  | 1.96 | [1.62; | 2.37] |  |  |  | 1.98 | [1.63; | 2.40] | 1.72 | [1.41; | 2.11] |
|  | Undefinable |  |  |  |  |  |  |  |  |  |  |  |  | 2.03 | [1.45; | 2.84] |  |  |  | 1.91 | [1.34; | 2.73] | 1.51 | [1.05; | 2.17] |
| Treatment | Intensive chemotherapy | | | |  |  |  |  |  |  |  |  |  |  |  |  | ref |  |  |  |  |  |  |  |  |
|  | Low intensive therapy | | | |  |  |  |  |  |  |  |  |  |  |  |  | 1.56 | [1.22; | 2.00] |  |  |  | 1.39 | [1.05; | 1.84] |
|  | Best supportive care | | | |  |  |  |  |  |  |  |  |  |  |  |  | 4.15 | [3.00; | 5.73] |  |  |  | 3.18 | [2.21; | 4.58] |
| **Time varying component** | | | | |  |  |  |  |  |  |  |  |  |  |  |  |  |  |  |  |  |  |  |  |  |
| Time * Performance status | | | | | 0.9998 [0.9997;0.9999] | | |  | | |  |  |  |  |  |  |  |  |  | 0.9999 [0.9998; 1.0000] | | | 0.9999 [0.9998; 1.0000] | | |
| Time * White blood cell counts | | | | |  |  |  |  |  |  | 0.9993 [0.9990; 0.9996] | | |  | | |  | | | 0.9993 [0.9989; 0.9996] | | | 0.9992 [0.9990; 0.9996] | | |
