# Supplementary material for: Are social inequalities in acute myeloid leukemia survival explained by differences in treatment utilization? Results from a French longitudinal observational study among older patients
Source: BMC Cancer. 2019 Sep 5;19:883. doi: 10.1186/s12885-019-6093-3 (PMC6729078; doi:10.1186/s12885-019-6093-3)
Supplement: Supplementary file 5 — Table S5. Step 2 Sensitivity analysis. Models of the association linking patients’ SEP to receiving Intensive Chemotherapy adjusted for each confounder, and fully adjusted. Generalized linear model with logit link function, adjusted odds ratios [95% Confidence Intervals], with missing data treated by multiple imputation (n = 685). (DOCX 20 kb) [file 12885_2019_6093_MOESM5_ESM.docx]

Table S5: Step 2 Sensitivity analysis. Models of the association linking patients’ SEP to receiving Intensitve Chemotherapy adjusted for each confounder, and fully adjusted. Generalized linear model with logit link function, adjusted odds ratios [95% Confidence Intervals], with missing data treated by multiple imputation (n=685)

|  | | Model 6.0 (M6.0) | | | Model 6.1 | | | Model 6.2 | | | Model 6.3 | | | Model 6.4 | | | Model 6.5 | | |
| --- | --- | --- | --- | --- | --- | --- | --- | --- | --- | --- | --- | --- | --- | --- | --- | --- | --- | --- | --- |
|  |  |  |  |  | M6.0 + perf. status | | | M6.0 + AML ontogeny | | | M6.0 + WBC | | | M6.0 + cytogen. Progn. | | | Fully adjusted | | |
|  |  | OR | [95% | CI] | OR | [95% | CI] | OR | [95% | CI] | OR | [95% | CI] |  |  |  | OR | [95% | CI] |
| Age | | 0.78 | [0.75; | 0.82] | 0.79 | [0.75; | 0.82] | 0.76 | [0.73; | 0.80] | 0.77 | [0.74; | 0.80] | 0.76 | [0.73; | 0.80] | 0.73 | [0.69; | 0.78] |
| Sex | Men | ref |  |  | ref |  |  | ref |  |  | ref |  |  |  |  |  | ref |  |  |
|  | Women | 0.65 | [0.40; | 1.05] | 0.65 | [0.40; | 1.05] | 0.63 | [0.37; | 1.05] | 0.74 | [0.44; | 1.22] | 0.83 | [0.49; | 1.40] | 0.92 | [0.51; | 1.66] |
| Patients’ SEP (quintile of deprivation score) | Q1 – least | ref |  |  | ref |  |  | Ref |  |  | ref |  |  |  |  |  | Ref |  |  |
|  | Q2 | 0.48 | [0.22; | 1.01] | 0.47 | [0.22; | 1.01] | 0.53 | [0.23; | 1.19] | 0.44 | [0.20; | 0.98] | 0.49 | [0.21; | 1.13] | 0.53 | [0.20; | 1.37] |
|  | Q3 | 0.99 | [0.50; | 1.97] | 0.97 | [0.48; | 1.95] | 1.08 | [0.52; | 2.27] | 0.99 | [0.48; | 2.04] | 0.81 | [0.38; | 1.71] | 0.90 | [0.38; | 2.14] |
|  | Q4 | 0.56 | [0.29; | 1.10] | 0.57 | [0.29; | 1.14] | 0.60 | [0.29; | 1.23] | 0.56 | [0.28; | 1.12] | 0.49 | [0.23; | 1.03] | 0.49 | [0.21; | 1.16] |
|  | Q5 – most | 0.43 | [0.20; | 0.95] | 0.46 | [0.21; | 1.02] | 0.61 | [0.25; | 1.44] | 0.42 | [0.18; | 0.96] | 0.48 | [0.20; | 1.13] | 0.62 | [0.22; | 1.72] |
| Charlson comorbidity index | 0 | ref |  |  | ref |  |  | ref |  |  | ref |  |  |  |  |  | ref |  |  |
|  | 1 | 0.62 | [0.34; | 1.15] | 0.65 | [0.35; | 1.20] | 0.66 | [0.34; | 1.28] | 0.59 | [0.31; | 1.12] | 0.71 | [0.37; | 1.38] | 0.76 | [0.36; | 1.60] |
|  | 2+ | 0.61 | [0.33; | 1.11] | 0.63 | [0.34; | 1.16] | 0.93 | [0.49; | 1.75] | 0.53 | [0.28; | 0.99] | 0.53 | [0.28; | 1.01] | 0.77 | [0.37; | 1.61] |
|  | Undefinable | 0.11 | [0.05; | 0.26] | 0.14 | [0.06; | 0.37] | 0.15 | [0.06; | 0.40] | 0.10 | [0.04; | 0.24] | 0.21 | [0.08; | 0.55] | 0.27 | [0.07; | 0.98] |
| Performance status | 0/1 |  |  |  | ref |  |  |  |  |  |  |  |  |  |  |  | Ref |  |  |
|  | 2 |  |  |  | 0.45 | [0.23; | 0.87] |  |  |  |  |  |  |  |  |  | 0.44 | [0.20; | 0.96] |
|  | 3/4 |  |  |  | 0.75 | [0.34; | 1.68] |  |  |  |  |  |  |  |  |  | 0.40 | [0.14; | 1.11] |
|  | Undefinable |  |  |  | 0.55 | [0.24; | 1.22] |  |  |  |  |  |  |  |  |  | 0.59 | [0.22; | 1.60] |
| AML ontogeny | AML de novo |  |  |  |  |  |  | ref |  |  |  |  |  |  |  |  | ref |  |  |
|  | Secondary (post MDS or post treatment) | | |  |  |  |  | 0.14 | [0.08; | 0.24] |  |  |  |  |  |  | 0.18 | [0.10; | 0.33] |
|  | Undefinable |  |  |  |  |  |  | Not estimated | | |  |  |  |  |  |  | Not estimated | | |
| White blood cell (WBS) counts (tercile) | Tercile 1 – low |  |  |  |  |  |  |  |  |  | Ref |  |  |  |  |  | ref |  |  |
|  | Terticle 2 – intermediate |  |  |  |  |  |  |  |  |  | 1.54 | [0.84; | 2.81] |  |  |  | 2.08 | [1.02; | 4.24] |
|  | Tercile 3 – high | |  |  |  |  |  |  |  |  | 5.80 | [3.09; | 10.88] |  |  |  | 6.95 | [3.30; | 14.63] |
|  | Undefinable | |  |  |  |  |  |  |  |  | Not estimated | | |  |  |  | Not estimated | | |
| Cytogenetic prognosis | Favorable/Intermediate |  |  |  |  |  |  |  |  |  |  |  |  |  |  |  |  |  |  |
|  | Unfavorable |  |  |  |  |  |  |  |  |  |  |  |  | 0.15 | [0.08; | 0.26] | 0.19 | [0.10; | 0.36] |
|  | Undefinable |  |  |  |  |  |  |  |  |  |  |  |  | Not estimated | | | Not estimated | | |
