# Supplementary material for: Are social inequalities in acute myeloid leukemia survival explained by differences in treatment utilization? Results from a French longitudinal observational study among older patients
Source: BMC Cancer. 2019 Sep 5;19:883. doi: 10.1186/s12885-019-6093-3 (PMC6729078; doi:10.1186/s12885-019-6093-3)
Supplement: Supplementary file 6 — Table S6. Step 3 Sensitivity analysis. Adjusted models of the association linking patients’ SEP to receiving non-intensive therapy among those who were not considered for intensive chemotherapy (n = 498). Generalized linear model with logit link function, adjusted odds ratios [95% Confidence Intervals] after treating missing data using multiple imputation. (DOCX 17 kb) [file 12885_2019_6093_MOESM6_ESM.docx]

Table S6: Step 3 Sensitivity analysis. Adjusted models of the association linking patients’ SEP to receiving non-intensive therapy among those who were not considered for intensive chemotherapy (n=498). Generalized linear model with logit link function, adjusted odds ratios [95% Confidence Intervals] after treating missing data using multiple imputation.

|  | | Model 7.0 (M7.0) | | | Model 7.1 | | | Model 7.2 | | | Model 7.3 | | |
| --- | --- | --- | --- | --- | --- | --- | --- | --- | --- | --- | --- | --- | --- |
|  |  |  |  |  | M7.0 + perf. status | | | M7.0 + WBC | | | Fully adjusted | | |
|  |  | OR | [95% | CI] | OR | [95% | CI] | OR | [95% | CI] | OR | [95% | CI] |
| Age | | 0.93 | [0.91; | 0.96] | 0.95 | [0.92; | 0.98] | 0.94 | [0.91; | 0.97] | 0.95 | [0.92; | 0.98] |
| Sex | Men | ref |  |  | ref |  |  | ref |  |  | ref |  |  |
|  | Women | 1.23 | [0.81; | 1.87] | 1.33 | [0.85; | 2.06] | 1.26 | [0.82; | 1.95] | 1.37 | [0.87; | 2.16] |
| Patients’ SEP (quintile of deprivation score) | Q1 – least | ref |  |  | ref |  |  | ref |  |  | ref |  |  |
|  | Q2 | 0.98 | [0.45; | 2.10] | 0.84 | [0.37; | 1.90] | 0.91 | [0.41; | 1.99] | 0.81 | [0.36; | 1.85] |
|  | Q3 | 1.16 | [0.58; | 2.32] | 1.12 | [0.54; | 2.35] | 1.16 | [0.56; | 2.39] | 1.16 | [0.54; | 2.48] |
|  | Q4 | 1.08 | [0.55; | 2.12] | 1.01 | [0.50; | 2.05] | 1.01 | [0.50; | 2.05] | 0.99 | [0.47; | 2.06] |
|  | Q5 – most | 1.12 | [0.56; | 2.23] | 1.13 | [0.55; | 2.31] | 1.14 | [0.56; | 2.33] | 1.16 | [0.55; | 2.44] |
| Charlson comorbidity index | 0 | ref |  |  | ref |  |  | ref |  |  | ref |  |  |
|  | 1 | 0.41 | [0.24; | 0.71] | 0.48 | [0.27; | 0.85] | 0.42 | [0.24; | 0.74] | 0.48 | [0.27; | 0.86] |
|  | 2+ | 0.43 | [0.24; | 0.76] | 0.51 | [0.28; | 0.94] | 0.41 | [0.23; | 0.74] | 0.48 | [0.26; | 0.90] |
|  | Undefinable | 0.08 | [0.05; | 0.15] | 0.12 | [0.07; | 0.23] | 0.10 | [0.05; | 0.17] | 0.13 | [0.07; | 0.25] |
| Performance status | 0/1 |  |  |  | ref |  |  |  |  |  | ref |  |  |
|  | 2 |  |  |  | 0.67 | [0.37; | 1.22] |  |  |  | 0.73 | [0.39; | 1.34] |
|  | 3/4 |  |  |  | 0.19 | [0.09; | 0.38] |  |  |  | 0.21 | [0.10; | 0.44] |
|  | Undefinable |  |  |  | 0.30 | [0.17; | 0.53] |  |  |  | 0.35 | [0.20; | 0.62] |
| White blood cell (WBS) counts (tercile) | Tercile 1 – low |  |  |  |  |  |  | ref |  |  | ref |  |  |
|  | Terticle 2 – intermediate |  |  |  |  |  |  | 0.72 | [0.41; | 1.25] | 0.83 | [0.47; | 1.47] |
|  | Tercile 3 – high | |  |  |  |  |  | 0.40 | [0.22; | 0.72] | 0.51 | [0.28; | 0.93] |
|  | Undefinable | |  |  |  |  |  | 0.09 | [0.02; | 0.47] | 0.11 | [0.02; | 0.58] |
